# Cancer stem cell niche models and contribution by mesenchymal stroma/stem cells

**DOI:** 10.1186/s12943-017-0595-x

**Published:** 2017-02-01

**Authors:** Catharina Melzer, Juliane von der Ohe, Hendrik Lehnert, Hendrik Ungefroren, Ralf Hass

**Affiliations:** 1 Biochemistry and Tumor Biology Lab, Department of Obstetrics and Gynecology, Hannover Medical School, Medical University Hannover, Carl-Neuberg-Str. 1, D – 30625 Hannover, Germany; 2 First Department of Medicine, University Hospital Schleswig-Holstein (UKSH), Campus Lübeck, Lübeck, Germany; 3 Department of General, Visceral-, Thoracic-Transplantation- and Pediatric Surgery, UKSH, Campus Kiel, Kiel, Germany

**Keywords:** Cancer stem cells, Tumor cell interaction, Tumor microenvironment, MSC, Retrodifferentiation

## Abstract

**Background:**

The initiation and progression of malignant tumors is driven by distinct subsets of tumor-initiating or cancer stem-like cells (CSCs) which develop therapy/apoptosis resistance and self-renewal capacity. In order to be able to eradicate these CSCs with novel classes of anti-cancer therapeutics, a better understanding of their biology and clinically-relevant traits is mandatory.

**Main body:**

Several requirements and functions of a CSC niche physiology are combined with current concepts for CSC generation such as development in a hierarchical tumor model, by stochastic processes, or via a retrodifferentiation program. Moreover, progressive adaptation of endothelial cells and recruited immune and stromal cells to the tumor site substantially contribute to generate a tumor growth-permissive environment resembling a CSC niche. Particular emphasis is put on the pivotal role of multipotent mesenchymal stroma/stem cells (MSCs) in supporting CSC development by various kinds of interaction and cell fusion to form hybrid tumor cells.

**Conclusion:**

A better knowledge of CSC niche physiology may increase the chances that cancer stemness-depleting interventions ultimately result in arrest of tumor growth and metastasis.

## Background

Various models are available for the generation of tumor initiating cells which subsequently give rise to neoplasias and malignant cancers including a hierarchical [1, 2] and a stochastic hypothesis [3, 4], a retrodifferentiation program [5–7] or MSC-tumor cell fusion to describe tumor initiation, heterogeneity, plasticity and progression [7–10].

### Tumor models

#### The hierarchical model

Tumor initiation of the hierarchical model starts within a normal stem cell niche (SCN) which regulates proliferation, apoptosis resistance and maintains stemness whereby a normal stem cell evades regulation resulting in an aberrant/tumorigenic stem-like cell, also known as cancer stem-like cell (CSC) [11, 12]. Besides the escape from regulation of normal stem cells, precursor or progenitor cells might evade stem cell niche regulation leading to cancer progenitor cells (CPC). Nonetheless, both CSCs and CPCs can develop within the stem cell niche to initiate tumor growth and give rise to impaired differentiated cell types with limited proliferative capacity. Accordingly, different CPCs can generate different subtypes of tumors [8, 13]. CSCs are characterized by their potential of self-renewal allowing them to drive tumor growth by generation of progeny with limited lifetime and proliferative capacity and by evasion of clonal exhaustion [14, 15]. Consequently, the hierarchical model delineates a non-tumorigenic cancer cell population with a distinct subset of CSCs featuring tumorigenic potential, regulating tumorigenesis and constituting the tumor as a heterogeneous population with distinct cell subsets in a particular tissue or organ [8, 16]. Since CSCs are assumed to be the cells of tumor origin, they are also designated as tumor-initiating cells (TICs) and may represent different populations in primary and metastatic tumors or with respect to the type of tumor. TICs have been identified in various primary tumors including human acute myeloid leukemia [2], breast cancer [1], human brain tumors [17], pancreatic cancer [18], ovarian cancer [19], human melanomas [20], prostate cancer [21], lung cancer [22], and human glioblastoma [23] among others. In addition, metastatic tumor tissue e.g. of breast [24, 25] or colon [26] also harbors TICs.

Examples of the hierarchical model have been shown in solid tumors such as breast cancer and in non-solid tumors such as acute myeloid leukemia [1, 2]. For instance, during in vivo application in immunodeficient mice only a subset of breast cancer cells developed tumorigenicity and could be separated from the non-tumorigenic population [1].

#### The stochastic model

The stochastic model represents a second feasibility to circumstantiate tumor initiation. In comparison to the hierarchical model, every tumor cell within the stochastic model is biologically homogenous with an equal probability to initiate, maintain and promote tumor growth whereby functionalities depend on both, extrinsic factors originating from the tumor microenvironment and intrinsic factors such as signaling pathways and levels of transcription factors [8, 27]. Tumorigenesis occurs from normal differentiated somatic cells that stochastically/randomly acquire oncogenic mutations resulting in hyperplasia, genomic instability, aberrant proliferation and expansion [3, 28].

Examples of the stochastic model can also be found in solid and non-solid tumors such as colorectal cancer, lung adenocarcinoma and lymphoblastic leukemias [29–32].

Whereas the stochastic model primarily addresses genetic heterogeneity without consideration of potential phenotypic variations within the genetically homogenous tumor cell population [8], the hierarchical model also represents a valuable model for a tumor relapse in those cancer patients where not all cancer cells and CSCs were successfully targeted during therapeutic approaches. Indeed, mouse xenografts of metastatic colon cancer demonstrated cancer origin and metastatic progression with features of both, the hierarchical model and the stochastic model for CSCs [26]. Therefore, these two models may provide supplementary information in view of a tumor cell switch between both models. A possible connection between the two models is represented by retrodifferentiation processes [7] to enable interconversion and correlation between the hierarchical and stochastic model (see 1.3). Thus, it is conceivable that tumor cells that arose according to the stochastic model retrodifferentiate into stem-like cells.

Consequently, both models of tumor initiation result in aberrant/tumorigenic stem-like cells which further promote tumor development and progression. However, little is known about the mechanism and the existence of a cancer stem cell niche (CSCN) for CSC generation and maintenance of tumor growth.

### Retrodifferentiation

Whereas tumor tissue harbors a variety of different cell populations including tumor cells in different states of development, one possibility of CSC development includes the hypothesis to be derived from neoplastic transformation during development or self-renewal of normal tissue-specific stem cells and to be primarily associated with solid tumors [33]. Alternatively, CSCs can develop by a retrodifferentiation process of differentiated tumor or tumor-associated cells to acquire self-renewal capacity and to maintain tumorigenicity [34, 35]. Retrodifferentiation is characterized by a reversion of all differentiated properties back to a stem-like phenotype including rejuvenation [36]. Consequently, retrodifferentiation extends the unidirectional view of cellular hierarchy to multi-directional possibilities of development, whereby retrodifferentiated and rejuvenated stem-like tumor cells exhibit the potential of self-renewal. Certain solid and non-solid in vitro tumor models were developed to study retrodifferentiation [7, 37]. Thus, induction of differentiation in a pheochromocytoma tumor cell line by nerve growth factor (NGF) was associated with a complete growth arrest and development of a sympathetic neuron-like phenotype by extension of neuritic processes similar to NGF-differentiated chromaffin cells. Molecular signaling events of this tumor cell differentiation involved NGF receptor-mediated phosphorylation of gp140trk and downstream signaling via the transcription factors c-Fos and EGR-1 for the induction of neuronal genes including transin, VGF-8 and voltage-gated sodium channels among others [38, 39]. Interruption of the receptor-activated signaling cascade e.g. by NGF removal reverted subsequent gene induction and the acquired neuronal functions and was accompanied by degeneration of the neurites. In parallel to necroptosis in some cells, the rest of the differentiated population reverted back to the pheochromocytoma tumor phenotype and regained proliferative capacity during this retrodifferentiation program [40].

Moreover, in a human myeloid leukemia model, phorbol ester-induced differentiation of U937 leukemia cells resulted in acquired adherence of cell cycle-arrested and differentiated monocyte/macrophage-like cells for several weeks. A decreasing threshold of phorbol ester or interference with the downstream signaling cascade of phorbol ester-activated protein kinase C interrupted transactivating processes via AP-1 (predominantly Jun/Fos) and NFκB and induced retrodifferentiation [41, 42]. This also promoted some apoptosis and necroptosis by decreasing the activity of poly-ADP-ribose polymerase-1 (PARP-1) which is important for DNA damage repair and PARP-1-mediated proteasomal degradation of oxidized and aberrant proteins [43–45]. Concomitant with the accumulation of these damage products and increasing formation of damage-associated molecular patterns (DAMPs), a subsequent retrodifferentiation process was induced in a majority of cells, whereby the differentiated cells lost all acquired macrophage-like properties and returned to a suspension growing leukemic phenotype with regained self-renewing capacity. These retrodifferentiated human cells are indistinguishable from undifferentiated leukemia cells and can repeatedly undergo such a phorbol ester-induced differentiation/retrodifferentiation cycle.

**Fig. 1:**
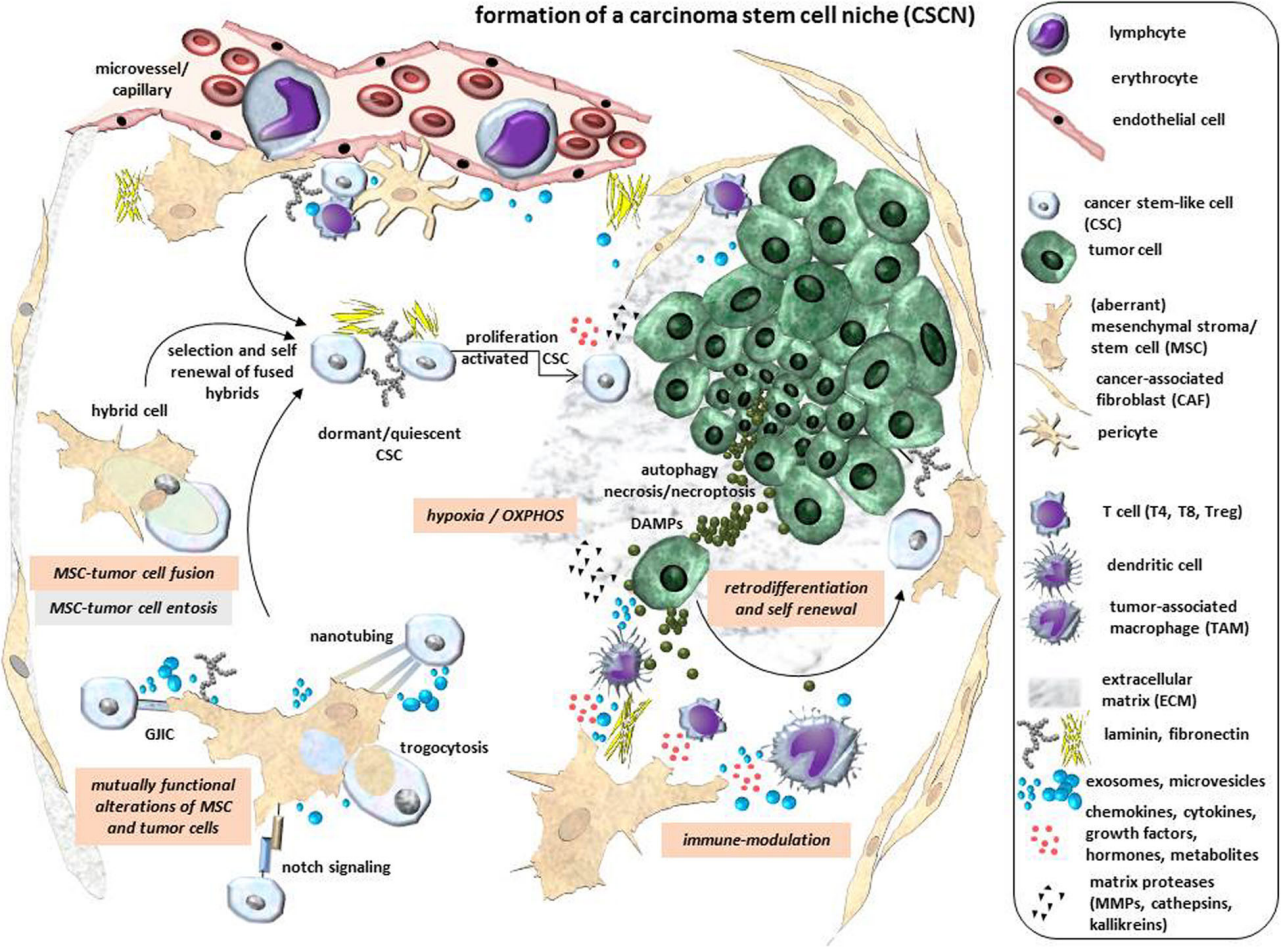
Hypothetical model for a CSCN compartment for CSC development. Due to oxygen and nutrient deprivation in a centralized localization of the tumor, starving tumor cells shift energy metabolism to enhanced anaerobic glycolysis with lactate accumulation and release whereby some tumor cells exhibit autophagy or undergo necroptosis by production of DAMPs. Interaction of DAMPs with adjacent tumor cells at oxygen-deprived hypoxic conditions and lactate-mediated low pH can induce retrodifferentiation and CSC development. Recruitment and activation of immune cells by DAMPs and the cytokine-mediated inflammatory environment is altered by immune-modulatory activities of cytokines-, chemokines- and exosomes-releasing MSC also accumulating at the inflammatory sites of the tumor. Release of mediators and exosomes by both, tumor cells and MSC can also mutually alter functionality of both cell types and induce CSC generation. Furthermore, MSC directly interact with tumor cells by various different mechanisms whereby close interactions at certain conditions result in entosis or hybrid cell formation via MSC – tumor cell fusion. Both mechanisms develop different kinds of hybrid cells which exhibit divergent functionalities during further tumor development. Subsequent selection processes of hybrid cells after MSC – tumor cell fusion contribute to CSC development. CSCs in perivascular regions can be kept in a dormant/quiescent state before cytokine/growth factor stimulation can activate re-entry into the proliferative cell cycle and self-renewal

**Fig. 2:**
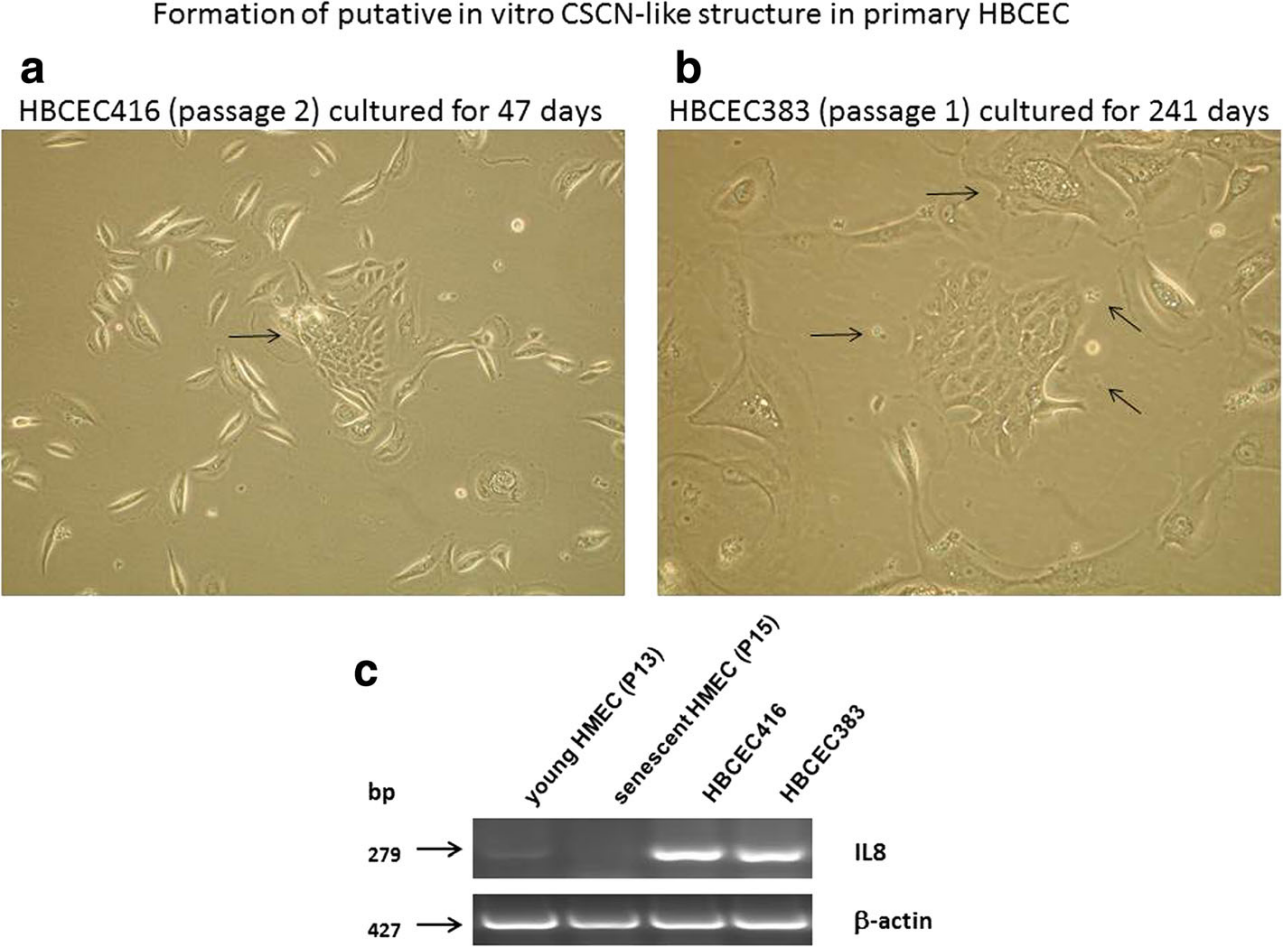
Formation of putative in vitro CSCN-like structures of primary human breast cancer-derived epithelial cells (HBCECs). Long-term cultivation of primary HBCEC416 (passage 2) for 47 days (**a**) and HBCEC383 (passage 1) for 241 days (**b**) [105] was associated with development of a central area with small proliferative active cells surrounded by a circle of larger growth-reduced and senescent cells demonstrating partial vesicle accumulation and release (arrows). Together with gradients of various soluble factors, these biological material-containing stimulatory vesicles may contribute to development of small-sized breast cancer stem-like cells and self-renewal. This is also substantiated by a significant expression of IL8 (PCR primer sense: 5′-AAAAAGCCACCGGAGCACT-3′; antisense: 5′-TTTCTGTGTTGGCGCAGTGT-3′; amplification product: 279 bp) in the corresponding HBCECs as compared to normal juvenile HMECs in P13 or growth-arrested and aged HMECs in P15 [74, 75] with β-actin as a control (**c**). Further supportive evidence is provided in breast and pancreatic cancer cells with IL8 expression by CSCs [102, 104, 106]

Together, these findings suggest that certain stimuli which may include damage products and DAMPs within a tumor cell population can establish a CSCN and contribute to a retrodifferentiation process to rejuvenate tumor cells to a more stem-like phenotype with enhanced self-renewal capacity (Fig. 1, Fig. 2a-c). Moreover, acquisition of tumor cell stemness via retrodifferentiation depends on a sensitive balance of timely available metabolite gradients and thresholds to enable the various steps of a retrograde development towards a CSC.

### MSC – tumor cell hybrids by entosis or fusion

A sensitive balance of timely available internal and external stimuli within a CSCN may also enable other modes of CSC development such as MSC-tumor cell fusion or entosis. Both types of interaction involve MSC as a potential cellular partner resulting in distinct functional hybrids. Although generally considered rare events, formation of hybrid cells via entosis or via fusion follow completely different mechanisms [46]. Entosis represents a form of cell-in-cell internalization mediated by the Rho-ROCK-actin/myosin pathway after loss of cell-matrix adhesion with subsequent release, cell division, or degradation of the target cell in the newly formed hybrid [47–49] which may contribute to the regulation of CSCs [46, 50]. Alternatively, tumor cell fusion depends on sensitive and balanced conditions such as hypoxic environment, low pH and appropriate membrane compatibility and the resulting tumor hybrid cells share genomic parts of both fusion partners while undergoing further post-fusion changes. In most hybrid cells, subsequent cell division is hampered by uncoordinated regulatory interactions of the two nuclei from the fused cells eventually resulting in cell death. Following a selection process with a loss of various chromosomes, however, some MSC-tumor cell fusion hybrids can re-establish a coordinated cell cycle progression by exhibiting CSC properties such as self-renewal capacity [51] (Fig. 1). Consequently, MSC-tumor cell entosis in contrast to fusion-derived hybrids between MSC and tumor cells develop different kinds of hybrid cell populations which most likely display divergent functionalities during further tumor development. Fusion of MSC with cells from different tumors including breast, ovarian, gastric and lung cancer has been demonstrated in vitro and in vivo [9, 52–54]. Moreover, human breast cancer can also fuse with normal breast epithelial cells [55]. Appearance of aneuploidy or polyploidy in human tumor cells with >46 chromosomes may result from aberrant/asymmetric cell division or previous cell fusion. Indeed, fusion of stem cells with other populations including terminally differentiated cells or somatic cancer cells has been discussed for recombination of DNA after nuclear fusion and reprogramming with potential contribution to tumor initiation suggesting the formation of CSCs [51, 56, 57].

## Conditions and requirements for the CSCN

The normal SCN harbors stem cells and is responsible for regulating stem cell maintenance, in particular the balance between self-renewal and differentiation. Moreover, the normal SCN represents a dynamic and complex compartment whereby additional components including endothelial, immune and stromal cells, extracellular matrix, cell adhesion molecules, soluble factors and microvesicles/exosomes contribute to an environment necessary for enabling both, self-renewal and the capability to differentiate [58]. Based on similarities between normal stem cells and CSCs such as the potential to self-renew, there is increasing evidence that CSCs also reside in similar niches, the CSCN, at the tumor site [11, 12, 59]. To better understand tumorigenesis and the concept of CSCs, appropriate models are helpful to elucidate conditions and requirements in a CSCN (Fig. 1).

Previous work described that stem cells reside in fixed compartments together with other cells determining stem cell behavior and regulating stem cell maintenance [60]. Thus, the CSCN may be regarded as defined compartment in which structural elements, soluble factors and cell-to-cell interactions with adjacent cell types of predominantly stromal origin contribute to cancer stem cell maintenance (Fig. 1).

One major prerequisite for tumor growth is the supply with nutrients and oxygen via blood vessels indicating the necessity of a CSCN localizing in the vicinity of vascular structures. Indeed, brain tumor stem cells have been reported to reside at perivascular regions [12]. In various stem cell niches, vascular cells have been attributed an important role in stem cell maintenance, e.g. in the bone marrow [61], adult hippocampus [62], the intestine and skin [63].

Besides neo-vascularization within the tumor microenvironment, the extracellular matrix (ECM) provides an important structural scaffold comprising fibrous proteins such as collagens, elastin, laminins, and fibronectin, globular proteins including the IgG superfamily integrins and cellular proteases, for instance MMPs, cathepsins and kallikreins for ECM remodeling [64]. During tumorigenesis, the ECM appears commonly dysregulated and disorganized [65, 66]. However, increasing evidence suggests, that ECM compounds are essential for stem cell niches. Stem cells have been shown to be closely associated with laminins surrounded by endothelial cells whereby progenitor cells were demonstrated to express the laminin receptor α6β1 integrin [67]. Inhibition of binding to laminin prevented adhesion to vascular endothelial cells thereby affecting proliferation. This is in concert with recent findings suggesting that adhesion to adjacent cells and extracellular matrix components contributes to the regulation of stem cell maintenance [68]. In the perivascular glioblastoma stem cell niche, laminin derived from non-stem tumor cells and tumor-associated endothelial cells affected tumor growth and CSC self-renewal capacity [69]. Moreover, laminin-111 in a three-dimensional cell culture system induced quiescence of breast epithelial cells by depletion of nuclear-associated actin [70, 71] (Fig. 1). Whereas the level of nuclear actin contributes to balance cell growth at least in breast tissue, the presence of laminin within the ECM likely would also display an important structural component of a CSCN.

When viewing a CSCN as a coordinated network of locally interacting cells (endothelial (precursor) cells, adipocytes, immune cells (T cells, Natural killer (NK) cells, dendritic cells (DC), macrophages) and mesenchymal cells (fibroblasts, vasculature-associated pericytes, MSC)) together with dynamic thresholds and gradients of soluble factors (exosomes and microvesicles, chemokines, cytokines, growth factors, hormones, metabolites) in a specific ECM environment (laminin, fibronectin, collagen, proteoglycans, etc.), then interference with this balanced homeostasis is predicted to alter CSC development (Fig. 1). Thus, ECM degradation and remodeling during tumor growth to enable tumor cell migration involves a plethora of cellular proteases including MMPs, cathepsins and kallikreins [72] which are also expressed by CSCs. For instance, glioblastoma CSCs express MMP-13 to enhance migration whereas knockdown of MMP-13 reduced migratory and invasive capacity of these CSCs [73]. Other matrix proteinases including MMP-1, MMP-7, and MMP-9 play important roles in normal and tumorigenic breast tissue remodeling and development [74–76]. Thus, following laminin-111 degradation by MMP-9 in the ECM, the tissue architecture of breast cells becomes lost and cell proliferation is enhanced [77]. Cathepsins also contribute to ECM degradation, whereby an additional function of cathepsins includes the maintenance of CSC self-renewal [78]. Down-modulation of cathepsin B (concomitant with the urokinase receptor (uPAR, CD87)) results in decreased expression of stem cell markers such as Sox2 and Nestin and reduces the glioma stem-like pool.

Human tissue kallikreins also belong to the family of serine proteinases that are involved in degradation of ECM components such as fibronectin, laminin and collagen [79, 80]. In ovarian cancer, overexpression of human kallikreins 4, 5, 6 and 7 accelerated tumor aggressiveness and invasiveness [81]. However, kallikreins might also act as ligands for proteinase-activated receptors (PARs), a class of G protein-coupled receptors that are activated by proteolytic cleavage [82]. PAR2 is activated by various kallikreins and can promote invasiveness and metastatic pathways in tumor cells either on its own [83] or by a crosstalk via TGF-β signaling, thereby enhancing the pro-migratory [84] and possibly pro-metastatic effects of this growth factor. More importantly, human kallikrein 3 also known as prostate-specific antigen (PSA) and used as prognostic tumor marker in prostate cancer diagnosis was more than 7-fold upregulated in CD133-positive prostate CSCs compared to other (CD133-negative) prostate cancer cells [85] supporting the concept within a CSCN that CSCs acquire increased migratory and metastatic potential.

Apart from distinct ECM components and appropriate restructure by distinct proteases that are required for a CSCN to promote CSC development, self-renewal and migration, adjacent cell types are also associated with a CSCN establishment via direct and indirect communication processes with tumor-derived cells to enable CSC development.

## Potential role of MSC in the maintenance of CSC/the CSCN

An important cell population during tumorigenesis is represented by MSC. These multipotent stromal cells are located predominantly at perivascular niches of nearly all human tissues and organs and display a plethora of functions including tissue repair, immunomodulation and stem cell homeostasis [86–89]. Subpopulations exhibiting different properties within MSC cultures demonstrated a heterogeneous stem cell entity [90]. During tumorigenesis, MSC are recruited to the inflammatory microenvironment of the tumor site [91]. Various studies have demonstrated interactions between MSC and cancer cells with support of CSC maintenance including breast, ovarian, lung and colon cancers [9, 52, 92–94]. In vivo studies revealed the impact of bone marrow-derived MSC on breast cancer stem-like cells by an accumulation of MSC and cytokine release within the breast tumor microenvironment which was associated with an increased number of CSCs [95]. Apart from the expression of specific surface markers, MSC are characterized by their ability to differentiate along the chondrogenic, osteogenic and adipogenic lineages [96] whereby also cross-germline differentiation capacity of MSC with cellular properties other than from mesodermal origin are discussed. At tumor sites, MSC can differentiate into cancer-associated fibroblasts (CAFs) which in turn favor tumor development [97, 98]. Co-culture experiments revealed the contribution of CAFs as feeder cells to supply stemness factors since CAFs from non-small cell lung carcinoma (NSCLC) patients promoted lung cancer stem-like cell growth. Conversely, removal of CAFs from the co-culture led to a down-modulation of stem cell markers such as Oct3/4 and Nanog followed by a partial differentiation of lung CSCs [99]. Moreover, sarcomas were hypothesized to originate from MSC by development of a CSC phenotype [50]. Furthermore, in vitro and in vivo glioma stem cells were capable to generate pericytes indicating an active role of CSCs to remodel their CSCN for additional vasculature and nutrient support [100]. In addition to MSC, CAFs and pericytes, immune cells have also been suggested to play a major role in CSCN maintenance, e.g. tumor-associated macrophages (TAMs) facilitated survival and growth of breast CSCs in vivo [101].

Regulation of CSC generation also involves a diverse range of soluble factors including cytokines, chemokines, growth factors, hormones, metabolites and further trophic molecules. Breast cancer stem-like cells which are characterized by low levels of CD24, high levels of CD44, and aldehyde dehydrogenase expression [1, 102, 103] have been suggested to express the IL8-binding chemokine receptor CXCR1. Neutralization of CXCR1 via a specific blocking antibody or small-molecule inhibitors decreased CSC populations and was accompanied by apoptosis/necroptosis of the cancer cell population indicating the requirement of IL8 signaling for CSC survival [104]. In vitro cultivation of human breast cancer-derived epithelial cells (HBCECs) [105] can develop CSCN-like structures which was also accompanied by IL8 expression in contrast to normal human mammary epithelial cells (HMECs) (Fig. 2a-c). Whereas HMEC culture eventually ends up in growth arrest and senescence [74, 75] long term cultivation of HBCEC populations maintains the capability to generate new proliferative active cancer cells (Fig. 2a and b). A potential IL8 production and corresponding signaling via CXCR1 has also been attributed to CSCs of pancreatic cancer [106].

Production and release of CCL5 by MSC has been suggested to activate corresponding receptors such as CCR5 on adjacent breast cancer cells thereby promoting altered breast cancer development and metastasis [107]. Moreover, autocrine CCL5-signaling via its receptors CCR1 and CCR3 accelerated migration and invasion of ovarian CSCs while either removal of CCL5 or blockade of CCR1 and CCR3 prevented their invasive potential [108]. Further soluble factors which interfere with CSC maintenance are microRNAs (miRs). For instance, miR-34 expression resulted in a reduced pancreatic TIC population [109] and exogenous miR-134 overexpression decreased human endometrial CSC migration [110].

### Direct communication of MSC with tumor cells as part of a CSCN

According to their recruitment to tumor sites associated with direct interactions of MSC with tumor cells, multipotent MSC may represent a major cellular component of a CSCN since various studies reported mutual acquisition of properties between both interaction partners which alter the original cell fate [9, 52].

Gap junctions enable the direct interaction between two neighboring cells, also known as gap junctional intercellular communication (GJIC). Thereby, each cell contributes equally to gap junction formation. Gap junction channels consist of hemichannels/connexons which in turn are composed of six connexin protein subunits that form a pore through the plasma membrane [111, 112]. In general, ions like Ca^2+^, small molecules such as microRNAs or second messenger are transported and exchanged via gap junctions allowing regulation of cell proliferation, differentiation and homeostasis maintenance [111, 113]. During co-culture with MSC, breast cancer cells acquired CD90 expression as a mesenchymal stem cell surface marker. Gap junction inhibitors decreased MSC-mediated CD90 acquisition of breast cancer cells indicating the involvement of GJIC in the process of cancer cell alteration [9]. Furthermore, GJIC has been reported in cancer cell dormancy. MiRs targeting CXCL12 were transferred via gap junctions from bone marrow stromal to breast cancer cells resulting in decreased CXCL12 levels and reduced proliferation thereby promoting cancer cell quiescence [114]. Moreover, bone marrow MSC-derived exosomes which include miR-23b can induce quiescence in bone marrow-associated breast cancer cells [115]. Dormancy/quiescence of breast cancer cells has also been attributed to interaction with the microvasculature, particularly endothelial cell-derived thrombospondin-1 whereas escape from dormancy and regained tumor cell proliferation is associated with sprouting neovasculature and availability of appropriate growth factors in the perivascular niche [116] (Fig. 1).

Whereas GJIC proceeds between two tightly adjacent cells, nanotubes are characterized by thin, F-actin rich structures which link interacting cells over longer distances. These dynamic cytoplasmic protrusions facilitate communication via exchange of various biological cargo including small molecules and organelles [117]. Notably, nanotubes enabled the transfer of mitochondria from bone marrow-derived MSC to breast cancer cells inducing increased oxidative phosphorylation (OXPHOS), proliferation and invasion of cancer cells [118]. Thus, nanotubes represent a crucial interaction tool for cancer cells to acquire altered cellular functions facilitating tumor survival, growth and expansion.

The Notch signaling pathway plays a crucial role in cellular processes including tissue repair, stem cell maintenance and regulation of immune cell functions [119]. There is increasing evidence that Notch signaling promotes pro-tumorigenic functions in solid tumors and is involved in cancer stem-like cell survival [120–122]. The Notch signaling cascade starts with ligand binding from the signal-sending cell to the notch receptor of the signal-receiving cell followed by cleavage of the receptors intracellular domain by a presenilin-γ-secretase. The cleavage domain translocates into the nucleus and activates downstream target genes by facilitating displacement of a transcriptional repressor [123]. Recent studies have identified MSC as signal-sending cell of Notch signaling whereas breast cancer cells received signals. Acquired expression of the MSC marker CD90 by breast cancer cells during co-culture was reduced by blocking of Notch signaling [9] suggesting a functional role of this pathway during cancer cell alteration. Additionally, CD90 has been proposed as marker for liver CSCs. In CD90-positive liver CSC featuring chemoresistance, migration, self-renewal, elevated invasiveness and metastasis, the Notch signaling pathway was activated. Conversely, inhibition of Notch signaling reduced migration, invasiveness and expression of stem cell-related genes further strengthening the importance of Notch signaling for CSCN maintenance [124].

Trogocytosis has been initially observed between immune cells as an active mechanism whereby lymphocytes extract surface molecules from antigen-presenting cells [125]. More recently, trogocytosis has been proposed as interaction mechanism by exchange of membrane patches and associated proteins between adjacent cells including MSC and cancer cells. Thus, ovarian tumor cells extracted membrane patches from stromal cells harboring multidrug resistance proteins thereby developing chemoresistance to platin and taxans [126]. Likewise, rare tumors of the small cell carcinoma of the ovary, hypercalcemic type (SCCOHT), demonstrated progressive chemo- and apoptosis resistance mediated by MSC [127].

Direct interaction and communication between MSC and tumor cells including GIJC, nanotube formation, Notch signaling, and trogocytosis may contribute to the generation of CSCs together with mutual exchange of distinct factors which alter properties of the involved cell populations. For example, cancer cell–derived interleukin1 can stimulate prostaglandin E2 secretion by MSC operating in an autocrine manner to further induce expression of cytokines by the MSC which in turn activate β-catenin signaling in the cancer cells in a paracrine fashion and formation of CSCs [128].

Together, these different types of direct interactions emphasize the importance and requirements of tumor-associated cells such as MSC within a CSCN to relay cellular properties that alter the original phenotype of tumor cells towards CSCs.

### Indirect communication of MSC with tumor cells

In addition to direct interactions altering CSC phenotype and function, indirect communication plays a pivotal role within CSCN. It involves both the release of soluble molecules such as metabolites and hormones and the exchange of microvesicles and exosomes [64].

In CSCN, metabolites including lactate, glutamine and keton bodies mutually reprogram metabolism of stromal stem cells and cancer cells favoring adaption of tumor cells to dynamic fluctuation of CSCN. Activation of CSCN homing CAFs by tumor cells leads to metabolic reprogramming of CAFs to a glycolytic phenotype meaning elevation of glucose uptake and elevation of lactate secretion serving as nutrient for adjacent cancer cells [129, 130]. On the one hand, lactate secretion increases acidity of CSCN resulting in higher ECM protease activity for migration and metastasis. On the other hand, lactate is taken up by cancer cells which reprograms their metabolism from glycolytic to respiratory mode (OXPHOS) maintaining cancer growth [131]. Indeed, osteosarcoma cells activate expression of lactate efflux receptors in MSC concomitant with accelerated expression of lactate influx receptors and lactate uptake in cancer cells. This metabolic shift increases ATP production and enhances migratory potential of osteosarcoma cells [132] indicating a necessity of acidification and metabolic reprogramming for increased tumor growth and progression. In addition to lactate, MSC deliver further nutrients such as ketone bodies and glutamine which can only be metabolized by OXPHOS fostering cancer growth [131] or arachidonic acid metabolites like prostaglandin E2 which modulates immune cells and protects lymphoblastic leukemia cells from cell death [133]. Moreover, prostaglandin E2- and cytokine-producing MSC can create a cancer stem cell niche together with other recruited cell populations to enable tumor progression [128].

Furthermore, hormones as soluble agents have been demonstrated to influence CSCs. For instance, progesterone induced the expansion of breast cancer stem-like cells [134].

Exosomes are characterized as homogeneous, 40 to 100 nm small endocytosed membrane particles which can be mutually exchanged by tumor cells and adjacent cell populations in the tumor microenvironment, particularly macrophages and MSC. These small particles contain a variety of proteins, lipids, functional RNAs and regulatory miRs [135, 136]. Although data are controversial concerning exosome function in tumorigenesis, there is predominant evidence that exosomes contribute to tumor growth whereby also tumor cell-derived exosomes play an important role [137, 138]. Recent work demonstrated the internalization of MSC-derived exosomes by breast and ovarian cancer cells resulting in new tumor cell properties and functions by acquisition of MMP2 and ecto-5′-nucleotidase (CD73, a MSC surface marker) activity, respectively, enabling increased potential to reorganize the tumor microenvironment [139]. Furthermore, MSC-derived exosomes enhanced proliferation and migration of breast cancer cells suggesting the involvement of Wnt signaling for elevated migration capacity [140]. In addition, certain miRs such as miR-222/223 from MSC-released exosomes promote dormancy/quiescence and drug resistance in a subset of breast cancer cells [141]. Intercellular communication between MSC and prostate cancer-derived exosomes activated the MSCs to differentiate into myofibroblasts whereby pro-angiogenic, pro-proliferative and pro-invasive functions were induced to facilitate tumor progression [142]. Tumor cell-derived exosomes in distinct organs also display distinct integrin expression patterns that can stimulate resident cells (macrophages, endothelial cells, MSC) to prepare a metastatic niche for tumor cells [143].

## Potential role of hypoxia, autophagy and DAMPs in CSC development

Although knowledge about CSCs originating from a CSCN is limited, the tumor microenvironment in which CSCs reside, provides a structural scaffold with various resident cancer-associated aberrant cell types which contribute to tumor growth and exchange soluble factors by mutual intercellular communications. Due to progressively increasing tumor cell growth and impaired vascularization, some tumor cells within the center of a solid tumor have limited access to nutrients. An impaired nutrient availability during expansion of the tumor size leads to hypoxic and more acidic conditions with starvation of the inner tumor cells eventually resulting in autophagy and necrosis/necroptosis [144] (see below).

Whereas such hypoxic and acidic milieu results from the imbalance between tumor cell proliferation and angiogenesis [145, 146], hypoxia represents one of the hallmarks of solid tumors influencing tumor development and progression [147] (Fig. 1).

Hypoxic signaling occurs via hypoxia inducible factors HIF-1 and HIF-2 that regulate cellular response to low oxygen and nutrient deficiency including activation of specific genes that control metabolism, angiogenesis, proliferation and differentiation [148]. Activation of angiogenesis increases tumor vascularization, however, tumor blood vessels feature abnormal pericyte coverage and leaky endothelial layers [149] and are thus unable to supply sufficient oxygen. Consequently, cancer cells adapt their metabolism to these environmental conditions also with altered energy metabolism. Normal cells primarily depend on energy storage and consumption via mitochondrial OXPHOS, however, cancer cells rely on glycolysis followed by increased lactate production which is supported by hypoxic conditions [150]. Similar effects are observed in MSC cultures, whereby hypoxic conditions were associated with induced HIF-1α expression and significantly elevated lactate production [151]. There is increasing evidence that cancer cells rely on both, glycolysis with lactate accumulation and OXPHOS whereby a shift between these two metabolic pathways indicates rapid adaptability of tumor cells to certain environmental conditions. Moreover, HIF-1α and HIF-2α expression were suggested to develop and maintain CSCs in gliomas [152] and in human neuroblastoma [153], respectively.

Together with the significant alterations in cellular metabolism, hypoxic conditions also mediate the activation of extracellular matrix proteases such as MT1-MMP and MMP-2 in mammary tumor cells [154] or gelatinase in distinct adenocarcinomas [155] which can restructure the ECM and accordingly, the architecture of a CSCN.

Furthermore, hypoxia induces epithelial-to-mesenchymal transition (EMT), a process required for metastasis, through activation of EMT transcription factors resulting in e.g. loss of E-cadherin [156, 157]. In general, EMT is characterized by alterations of epithelial-like cell properties towards a mesenchymal phenotype including downregulation of E-cadherin for loss of cell polarity, secretion of proteases for ECM degradation and an increase in mesenchymal marker expression for accelerated migration and invasiveness [158–160]. Cancer cells undergoing EMT have been demonstrated to acquire mesenchymal cell traits resulting in mesenchymal-like migration patterns of cancer cells through tumor microenvironment. This mesenchymal migration type is characterized by protease-dependency to facilitate ECM degradation via MMPs, cathepsins and kallikreins and to enhance movement through the ECM [72, 161, 162]. An EMT program induced by TGF-β is associated with the acquisition of stem cell traits, proliferation arrest and enhanced resistance to apoptotic stimuli including anti-cancer drugs (chemoresistance). Recent data in pancreatic ductal adenocarcinoma cells in vitro suggested that TGF-β1 induced the generation of CSC-like cells with clonogenic potential and that this process can be efficiently inhibited with the anti-cancer drug dasatinib (BMS-354825, Spryce) [163].

Following hypoxia and EMT, cancer cells can escape the primary tumor niche and migrate and disseminate to distant organs [164, 165].

Besides the contribution of hypoxic conditions to metastasis, low pH/acidic conditions as a result of lactate release from increased anaerobic glycolysis of tumor cells may favor metastasis as well. Acidic conditions are proposed to activate proteases such as cathepsins which in turn degrade ECM for tumor invasion [166–168]. Also, acidic stress favors the development of CSCs in gliomas [169].

Hypoxic and more acidic conditions in the inner part of a tumor are often accompanied by starvation and reduced tumor cell viability, Enhanced cell death of centrally located tumor cells by progressive nutrient deficiency, starvation and low oxygen levels can involve three main mechanisms: apoptosis, autophagy and necrosis/necroptosis. Apoptosis is a highly regulated cell death program that can be triggered by both extrinsic and intrinsic stimuli after induction in consequence of inevitable cell stress [170, 171]. However, many cancer cells and particularly those with a partial EMT phenotype including CSCs exhibit resistance to apoptosis [172] since in a hypoxic environment, expression of pro-apoptotic members of the Bcl-2 family is decreased while protein levels of anti-apoptotic mediators such as Bcl-xL are increased [173, 174]. This EMT-mediated loss in apoptosis sensitivity partially accounts for a chemoresistant phenotype. Autophagy is a well-regulated catabolic process that usually exerts pro-survival functions via lysosome-mediated degradation of intracellular molecules that provides energy needed during starvation or cellular stress [175]. Accordingly, autophagy plays an important supportive role in cancer development. Indeed, autophagy has been shown to promote survival of disseminating, dormant/quiescent and stem-like tumor cells and to be upregulated during metastasis [176]. These stem-like tumor cells can represent a heterogeneous population e.g. by subclones which carry mutations of known oncogenic potential but do not exhibit any signs of malignancy for long time and are therefore distinguished as “neoplastic stem cells” [177]. An enhanced contribution of autophagy to CSC activation has also been demonstrated in breast cancer cells by increased regulation of CD24^low^/CD44^high^ breast cancer stem-like cells [178]. Conversely, inhibition of autophagy in pancreatic tumor cells was associated with reduced activity of CSCs [179] further substantiating an important role of autophagy in regulating CSC functionality.

Necrosis depicts another process of cell death characterized as random, accidental and unregulated [180]. Nonetheless, regulated, programmed necrosis in tumor cells has been observed and termed necroptosis for controlled cell death [181]. Apoptotic, autophagic and necrotic/necroptotic cells within the tumor microenvironment release damage-associated molecular patterns (DAMPs) which serve as danger signals and are primarily recognized by pattern recognition receptors (PRRs) such as toll-like receptors [182] (Fig. 1). DAMPs are found in all stressed cells and are delineated as a large group of unrelated mediators including S100 proteins, ATP, heat shock proteins, hyaluronan, HMGB1 (high mobility group box 1), and calcireticulin [183]. Particularly the DAMP-associated protein HMGB1 has been suggested to promote cancer progression in malignant mesothelioma also evidenced by elevated serum levels of malignant mesothelioma patients which indicates a supportive role of DAMPs for CSC functions [184].

The release of DAMPs initiates an innate and adaptive immune response attracting immune cells such as DC, NK cells, macrophages and regulatory T cells (Tregs) [182] (Fig. 1). Although inflammation induces anti-tumor signaling which successfully eliminates the tumor cells, opposite effects facilitate tumorigenesis due to failure of an effective immune response and escape of some tumor cells from immune surveillance which results in DAMP-mediated tumor progression [183]. Indeed, glioblastoma cancer progression was associated with ineffective response of CSCs to DAMPs partially due to a decreased toll-like receptor expression and thereby, DAMPs contribute to CSC maintenance [185].

Reduced immune response to tumor cells can also be mediated by MSC which are recruited to tumor sites due to the inflammatory microenvironment (Fig. 1). Overall, MSC interact with a variety of immune cells and exhibit immune-modulatory functions. They suppress the cytotoxicity potential of NK cells or inhibit T cell activation by altering immune cell functions and favoring immune suppression [91]. Recent findings substantiated the anti-proliferative effects of MSC on T lymphocytes by expression of nitric oxide synthase and production of nitric oxide metabolites [186]. Moreover, MSC can regulate immune competence by release of IL-10 or by producing the enzyme indoleamine-2,3-dioxygenase (IDO) associated with induction of tolerance and a shift from Th1 to Th2 immune response. Furthermore, Tregs are severely affected by DAMPs such as adenosine and prostaglandin E2 [187, 188] and can interact with other immune cells leading to limited anti-tumor immunity [189].

Macrophages (M1) contribute to tumor destruction via IFNγ activation followed by production of type 1 cytokines and chemokines. Conversely, activation of M2 macrophages via MSC promotes tumorigenesis by production of type 2 cytokines and chemokines strengthening the dual role of macrophages depending on the phenotype and activation status. During progressive adaption to the tumor microenvironment, TAMs represent a further macrophage phenotype that triggers tumor development through support of angiogenesis and ECM remodeling [190]. Consequently, even though inflammation at tumor sites induces anti-tumor responses, attracted MSC alter immune cell functions and favor an immunosuppressive microenvironment with reduced immune surveillance which contributes to CSC development and promotion of tumor growth.

Together, the cascade of hypoxic conditions and low nutrient supply accompanied by limited apoptosis, autophagy and necrosis/necroptosis followed by release of DAMPs evokes an inflammatory microenvironment which is modulated by interacting MSC. These mechanisms which are also influenced by protease activities and subsequent ECM modulation interfere with the dynamic and sensitive equilibrium of the CSCN which can critically alter the amount of CSCs affecting clinical outcomes and patient prognoses [191].

## Conclusions

The presence of a CSC population as part of a heterogeneous tumor entity [192] is suggested with following functions: 1) cancer maintenance by self-renewal capacity; 2) differentiation and development capacity; 3) chemo/apoptosis resistance; 4) escape from immune surveillance. CSCs can evolve from normal SCNs, from primary tumors, from metastases with disseminated tumor cells after EMT, from cell fusion following subsequent selection, and/or from a retrodifferentiation process among others. Generation of CSCs requires a multistep cascade of development including genetic and/or epigenetic changes. CSC maintenance/protection in a dormant/quiescent state within a specialized microenvironment and activation by cytokines/growth factors for cell cycle reentry and tumor growth (relapse) remains a matter of debate among alternative hypotheses and models of a CSCN.

According to metabolic alterations and functional interference with the requirements of a carefully balanced factor homeostasis for CSC generation, the sensitive maintenance of a CSCN is likely subject to changes. Such CSCN structures can be disabled at certain sites of the tumor and newly established at more favorable places within the tumor suggesting multiple and simultaneous possibilities for CSCNs with appropriate turnover. A potential CSCN turnover may depend on the stability of the environment. For example, CSCNs of tumor metastases in the bone marrow are more protected and stabilized in the spongy bone cavities as compared to CSCNs in more metabolically-exposed tissues such as primary organ-associated tumor tissues or lymph node metastases. Nevertheless, the dynamic generation and changes of CSCs within the plasticity of tumor tissues and the continuously functional alterations/adaptations of developing and metastasizing tumor cells by loss of distinct functions and/or acquisition of new properties represent the real challenge of a successful tumor therapy.

## Abbreviations

CAF: Cancer-associated fibroblast
CPC: Cancer progenitor cell
CSC: Cancer stem-like cell
CSCN: Cancer stem cell niche
DAMP: Damage-associated molecular pattern
DC: Dendritic cell
ECM: Extracellular matrix
EMT: Epithelial-mesenchymal transition
GJIC: Gap junctional intercellular communication
HBCEC: Human breast cancer-derived epithelial cells
HMEC: Human mammary epithelial cells
HMGB1: High mobility group box 1
miR: MicroRNA
MMP: Matrix metalloproteinase
MSC: Mesenchymal stroma/stem cell
NK: Natural killer cell
OXPHOS: Oxidative phosphorylation
PRR: Pattern recognition receptor
SCN: Stem cell niche
TAM: Tumor-associated macrophage
TIC: Tumor-initiating cell
Treg: Regulatory T cell
